# Surface morphology data of tantalum coatings obtained by electrospark alloying

**DOI:** 10.1016/j.dib.2018.09.028

**Published:** 2018-09-14

**Authors:** Marina Fomina, Vladimir Koshuro, Vyacheslav Papshev, Igor Rodionov, Aleksandr Fomin

**Affiliations:** a Yuri Gagarin State Technical University of Saratov, 410054 Saratov, Russia; b TAV Dental, 2283202 Shlomi, Israel

## Abstract

The article presents the data of scanning electron microscopy of the surface morphology of tantalum coatings produced by electrospark alloying. To perform the statistical analysis of open porosity and morphological parameters of the coatings, raw digital images of the structure were studied. In this case, “AGPM” software for the analysis of geometric parameters of microobjects was used. When processing digital images of the surface morphology, open porosity, total quantity, average size and dispersion of the structural elements (particles and pores) were determined, and typical distribution patterns of structural elements were obtained from linear dimensions.

**Specifications table**

**Table:** 

**Subject area**	*Engineering*
**More specific subject area**	*Material science, Surface science, Coating technology*
**Type of data**	*Image (SEM)*
**How data was acquired**	*SEM. Scanning electron microscope "MIRA II LMU" (TESCAN, Czech Republic)*
**Data format**	*Raw and analyzed*
**Experimental factors**	*Test samples were made of commercially pure titanium VT1-00 (Grade 1 analog) [1] and titanium alloy VT16 (Ti - 82.89–89.7 wt.%, Al - 1.8–3.8 wt.%, V - 4.0–5.5 wt.%, Mo - 4.5–6.5 wt.%, and other: Fe - <0.25 wt.%, C - <0.1 wt.%, Si - <0.15 wt.%, N - <0.05 wt.%, Zr - <0.3 wt.%, O - <0.15 wt.%, H - <0.015 wt.%.) [2]. The surface preparation of the samples included fine turning (Ra = 1.6–3.2 µm) and cleaning in ethanol. Further, tantalum coatings were obtained on the surface of the samples by the method of electrospark alloying (ESA) at 3 operating current levels: 1.0 ± 0.2 A, 1.75 ± 0.25 A and 2.5 ± 0.3 A. At the same time, the pulse energy varied from 2.2 to 5.5 J. Other parameters of ESA remained unchanged: oscillation frequency of the vibrator – 100 ms, treatment speed – 175 ± 5 mm*^*2*^*/min.*
**Experimental features**	*The coatings were obtained by ESA using "EFI-46A" (Moldova). Open porosity and morphological parameters of the coatings were studied using "AGPM" software for the analysis of geometric parameters of microobjects. The following parameters were determined: open porosity, average particle and pore sizes, particle and pore particle size distribution graphs, total number of particles and pores, and dispersion.*
**Data source location**	*Yuri Gagarin State Technical University of Saratov, Laboratory "Electrophysical Processes and Technologies", Saratov, Russia*
**Data accessibility**	*Data is within this article*

**Value of the data**

• The experimental data and statistical estimation presented in this article can be used to provide a basis for morphological analysis of the surface of porous metal coatings, in particular the tantalum ones, obtained by electrospark alloying.
• The developed method for analyzing the parameters of the surface morphology of coatings has a high statistical significance, since it takes into account the parameters of all the particle and pore size modes in the digital image field.
• The processing of digital images using “AGPM” software has a binding to the linear dimensions (the frame width) of the structure under study; it involves the conversion of the polychromatic image into the black and white one, where light areas correspond to particles, e.g. formed coating microparticles (splats), grains and relief protrusions, and dark ones - to pores, including other cavities and cracks.
• The proposed analysis of digital images of the surface can be used for statistical processing of the structure parameters of porous materials and coatings, e.g. obtained by plasma spraying, oxidation, etc.

## 1 Data

The data were obtained using SEM of tantalum coatings formed on titanium samples by the ESA method. The surface morphology parameters of the coatings were determined using the statistical processing of digital images in "AGPM" software. For each sample, statistical values were determined for micro-sized particles and open pores of tantalum coatings.

## 2 Experimental design, materials and methods

Test samples were made of commercially pure titanium VT1-00 (Grade 1 analog) [1] and titanium alloy VT16 (Ti - 82.9–89.7 wt.%, Al - 1.8–3.8 wt.%, V - 4.0–5.5 wt.%, Mo - 4.5–6.5 wt.%, and other: Fe - <0.25 wt.%, C - <0.1 wt.%, Si - <0.15 wt.%, N - <0.05 wt.%, Zr - <0.3 wt.%, O - <0.15 wt.%, H - <0.015 wt.%.) [2]. The structure and properties of the selected titanium alloys were presented in [3].

The surface preparation of the samples included fine turning (Ra = 1.6–3.2 µm) and cleaning in ethanol. Further, tantalum coatings were obtained on the surface of titanium samples by ESA (“EFI-46A”) at 3 operating current levels: 1.0 ± 0.2 A, 1.75 ± 0.25 A and 2.5 ± 0.3 A, while the pulse energy varied from 2.2 to 5.5 J. Other parameters of ESA remained unchanged: the oscillation frequency of the vibrator was 100 ms, the treatment speed equaled 175 ± 5 mm^2^/min.

The images showing the surface morphology of tantalum coatings were obtained using SEM at a magnification of ×1000 and frame width of 331 µm (for the detection of micro-sized parameters of particles and open pores), accelerating voltage of 30 kV in the “SE” (secondary electrons) mode.

**Fig. 1:**
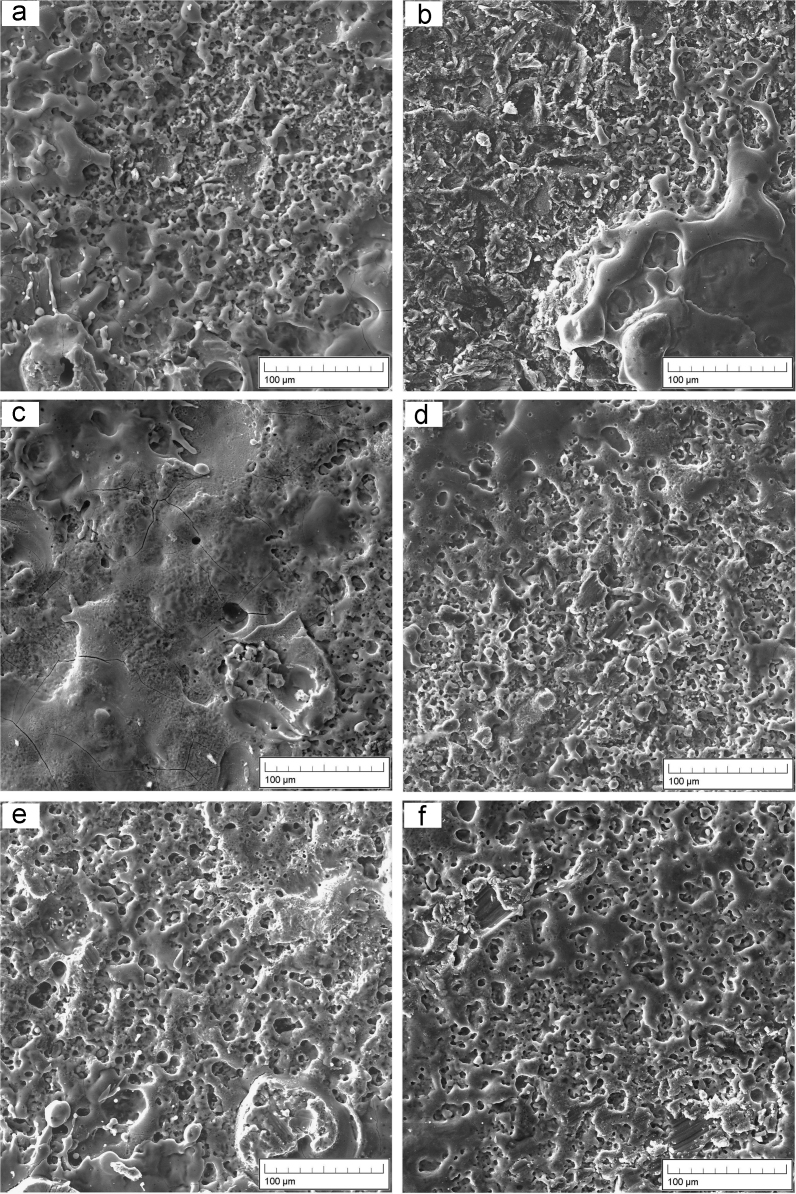
SEM of tantalum coatings obtained by ESA on VT1-00 (a,c,e) and VT16 (b,d,f) at different current levels: a,b – 1.0 ± 0.2 A; c,d – 1.75 ± 0.25 A; e,f – 2.5 ± 0.3 A (magnification × 1000).

**Fig. 2:**
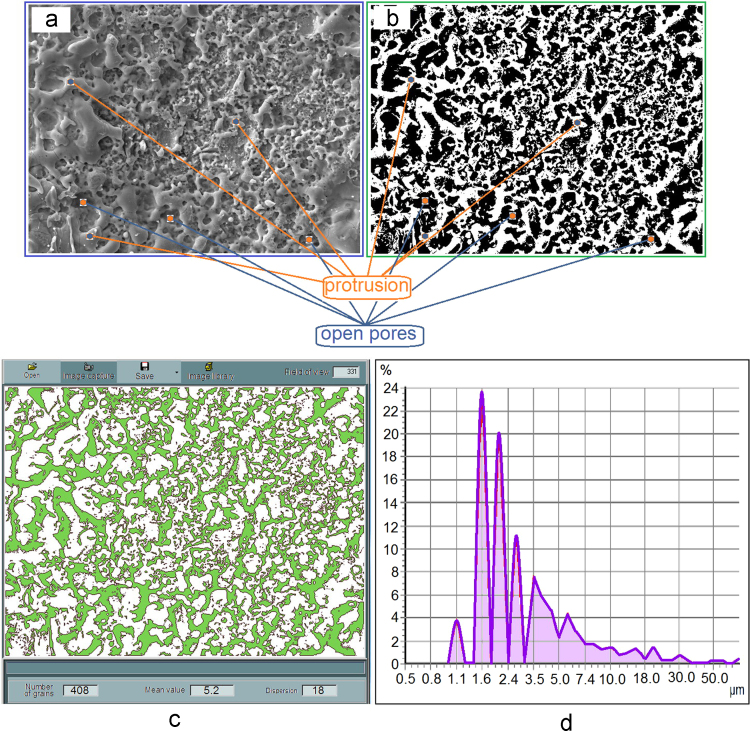
Stages of the data processing: a – selection of rectangular areas; b – conversion of the original image into a black and white one; c – statistical analysis of the image and production of the output data; d – graph of the microparticles distribution in size.

For the statistical evaluation of the morphological parameters, a sequence of digital image processing was developed. The main stages of the data processing included: 1. obtaining digital images of the morphology with the selected ESA modes (Fig. 1); 2. selection of rectangular areas (ratio width: height = 12:9) for the subsequent analysis and statistical processing of morphological parameters (Fig. 2a)*;* 3. entering data on the frame width of a rectangular image; 4. loading the image into "AGPM" software for analyzing the geometric parameters of micro-objects; 5. conversion of the original image into a black and white one (Fig. 2b); 6. calculation of porosity (black areas corresponded to open pores or cavities); 6. statistical analysis of the image and production of the output data, namely the average size of microparticles (or micropores in inverted images), the total number of microparticles (or micropores) and dispersion (Fig. 2c), and graphs showing the microparticles distribution (or micropores) in size (Fig. 2d)*.*

**Fig. 3:**
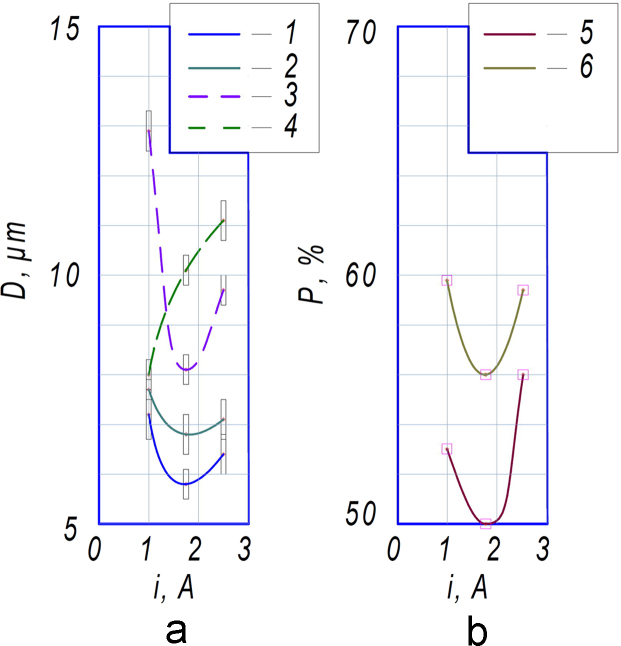
Dependencies of the mean values of parameter D (a) and porosity P (b) on the current j for VT1-00 (1,2,5) and VT16 (3,4,6): 1,3 – for microparticles; 2,4 – for micropores; 5,6 – for the porosity.

Table 1 summarizes the compiled data for all images of the morphology of tantalum coatings obtained by ESA that contain the initial data (materials substrate / working current, field of view / element type / structure type), output parameters (porosity; mean value of parameter; number of structure elements; dispersion) and a confidence interval. Mean values of parameter D (Fig. 3a), e.g. mean value of microparticles and micropores, with confidence interval and porosity P (Fig. 3b) are summarized in the graphs.

**Table 1:** Micro-sized parameters of the morphology of tantalum coatings obtained by ESA.

No.	Working current	Element type	Mean value of parameter	Number of structure elements	Dispersion	Confidence interval	Porosity
–	A	n.u.	µm	unit	µm^2^	µm	%
Materials substrate titanium VT1-00							
1-1	1.0 ± 0.2	Protrusions	7.2	408	24.3	0.5	–
1-2	1.0 ± 0.2	Open pores	12.9	494	24.1	0.4	53
2-1	1.75 ± 0.25	Protrusions	5.8	673	19.1	0.3	–
2-2	1.75 ± 0.25	Open pores	8.1	946	16.6	0.3	50
3-1	2.5 ± 0.3	Protrusions	6.4	538	21.0	0.4	–
3-2	2.5 ± 0.3	Open pores	9.7	767	18.2	0.3	56

Materials substrate titanium alloy VT16							
1-1	1.0 ± 0.2	Protrusions	7.7	1062	18.4	0.2	–
1-2	1.0 ± 0.2	Open pores	8.0	734	21.2	0.3	59
2-1	1.75 ± 0.25	Protrusions	6.8	638	20.8	0.4	–
2-2	1.75 ± 0.25	Open pores	10.1	753	18.6	0.3	56
3-1	2.5 ± 0.3	Protrusions	7.1	563	21.7	0.4	–
3-2	2.5 ± 0.3	Open pores	11.1	651	22.2	0.4	58
