# Supplementary material for: Surface morphology data of tantalum coatings obtained by electrospark alloying
Source: Data Brief. 2018 Sep 14;20:1409–14. doi: 10.1016/j.dib.2018.09.028 (PMC6148842; doi:10.1016/j.dib.2018.09.028)
Supplement: Supplementary file 1 — Supplementary material [file mmc1.pdf]

Conflicts of Interest Statement

Manuscript title: Surface morphology data of tantalum coatings obtained by electrospark alloying

The authors whose names are listed immediately below certify that they have NO affiliations with or involvement in any organization or entity with any financial interest (such as honoraria; educational grants; participation in speakers' bureaus; membership, employment, consultancies, stock ownership, or other equity interest; and expert testimony or patent-licensing arrangements), or non-financial interest (such as personal or professional relationships, affiliations, knowledge or beliefs) in the subject matter or materials discussed in this manuscript.

Author names:

___________________ / Aleksandr Fomin

24.07.2018

Corresponding author signs this document on behalf of all the authors of the submitted manuscript.
